# Capacity for Absorption of Water-Soluble Secondary Metabolites Greater in Birds than in Rodents

**DOI:** 10.1371/journal.pone.0032417

**Published:** 2012-02-28

**Authors:** William H. Karasov, Enrique Caviedes-Vidal, Bradley Hartman Bakken, Ido Izhaki, Michal Samuni-Blank, Zeev Arad

**Affiliations:** 1 Department of Forest and Wildlife Ecology, Russell Laboratories, University of Wisconsin-Madison, Madison, Wisconsin, United States of America; 2 Departamento de Bioquímica y Ciencias Biológicas, Universidad Nacional de San Luis, San Luis, Argentina; 3 Instituto Multidisciplinario de Investigaciones Biológicas de San Luis, Consejo Nacional de Investigaciones Científicas y Técnicas, Chacabuco, San Luis, Argentina; 4 Department of Evolutionary and Environmental Biology, University of Haifa, Haifa, Israel; 5 Department of Biology, Technion, Haifa, Israel; University of Western Ontario, Canada

## Abstract

Plant secondary metabolites (SMs) are pervasive in animal foods and potentially influence feeding behavior, interspecies interactions, and the distribution and abundance of animals. Some of the major classes of naturally occurring SMs in plants include many water-soluble compounds in the molecular size range that could cross the intestinal epithelium via the paracellular space by diffusion or solvent drag. There are differences among species in paracellular permeability. Using Middle Eastern rodent and avian consumers of fruits containing SMs, we tested the hypothesis that avian species would have significantly higher paracellular permeability than rodent species. Permeability in intact animals was assessed using standard pharmacological methodology to measure absorption of two radiolabeled, inert, neutral water-soluble probes that do not interact with intestinal nutrient transporters, L-arabinose (M_r_ = 150.1 Da) and lactulose (M_r_ = 342.3 Da). We also measured absorption of labeled 3-O-methyl-D-glucose (3OMD-glucose; M_r_ = 194.2 Da), which is a nonmetabolized analogue of D-glucose that is passively absorbed through the paracellular space but also transported across the enterocyte membranes. Most glucose was absorbed by all species, but arabinose fractional absorption (*f*) was nearly three times higher in birds (1.03±0.17, n = 15 in two species) compared to rodents (0.37±0.06, n = 10 in two species) (P<0.001). Surprisingly, the apparent rates of absorption in birds of arabinose exceeded those of 3OMD-glucose. Our findings are in agreement with previous work showing that the paracellular pathway is more prominent in birds relative to nonflying mammals, and suggests that birds may be challenged by greater absorption of water-soluble, dietary SMs. The increased expression of the paracellular pathway in birds hints at a tradeoff: the free energy birds gain by absorbing water-soluble nutrients passively may be offset by the metabolic demands placed on them to eliminate concomitantly absorbed SMs.

## Introduction

Secondary metabolites (SMs) are compounds produced and/or sequestered by plants and animals that do not appear to play a major role in their primary nutritional or regulatory metabolism. Their functions include communication, attraction, or defense against herbivores, predators, pathogens, and competitors [1]. SMs are so pervasive that arguably they occur in at least some foods consumed by most terrestrial vertebrates, and potentially they can influence feeding behavior, interspecies interactions, and the distribution and abundance of animals [2]. However, their impacts are not always general but may depend on particular digestive features and/or counter-responses of consumers.

Lipophilic SMs are anticipated to permeate intestinal cell (enterocyte) membranes passively at rates positively related to their octanol or oil∶water partition coefficients [3], although their rates might be decreased by complexing proteins [4], [5], physical barriers [6], [7], [8], and membrane transporters that bind them and export them back into the lumen [9], [10], [11]. It is to be expected that water-soluble SMs will not permeate the intestinal cell membrane because of its lipid bilayer, although it is theoretically possible that some SMs may bind to and be transported by membrane transporters (e.g., a rare example is [12]). Nonetheless, water-soluble SMs could be absorbed across the intestinal epithelium through paracellular spaces between adjacent enterocytes by diffusion or solvent drag [13]. The tight junctions adjoining adjacent enterocytes have selective permeability, discriminating among solutes by charge and size [14]. In both eutherian mammal and avian species the paracellular route of passive absorption of water-soluble compounds has been visualized by either autoradiography [15] or confocal laser microscopy [16], [17]. Intestinal paracellular absorption in mammals and birds appears to be qualitatively similar in regards to molecular size selectivity, as characterized using a series of non-electrolyte water-soluble probes that differ in molecular dimension [18], [19], and in charge selectivity as characterized using relatively inert charged peptides [20], [21]. Hence, some of the water-soluble SMs that are not too large in molecular size will have access to the paracellular pathway. Some of the major classes of naturally occurring SMs in plants, such as alkaloids and phenolics, include many water-soluble compounds in the molecular size range that could access the paracellular space [22]. Nicotine, for example, has a MW of 162 Da, its cationic forms are water soluble, and it was found to be absorbed by the paracellular pathway in cell culture (TR146 cells) [23].

There are differences among species in paracellular permeability [20], [24], [25], which leads to an expectation that absorption of water-soluble SMs could also differ among species. Previously, it was proposed that small birds (<500 g) generally have higher paracellular permeability than similar sized nonflying mammals, partly in compensation for having smaller intestines with less absorptive surface area [26]. However, that analysis was based on a disproportionate number of avian species in the small size range <150 g (*n* = 5 avian species *vs.* 1 mammal, the laboratory mouse). In this study we add substantially to the comparative data set at this smaller size range.

Using Middle Eastern rodent and avian consumers of fruits containing SMs, we tested for differences in paracellular absorption. We chose our species from the desert ecosystem in Israel. Tristram's grackle (*Onychognathus tristramii*, Sturnidae) and Yellow-vented bulbuls (*Pycnonotus xanthopygos*, Pycnonotidae), are two main avian seed dispersers [27], [28], and Common/Egyptian spiny mouse (*Acomys cahirinus*, Muridae) and Golden spiny mouse (*Acomys russatus*, Muridae) are two main seed predators (A. Haim, pers. comm.). We predicted the avian species would have significantly higher paracellular permeability than the two rodent species. Permeability was assessed by measuring absorption by intact animals of two relatively inert, neutral water-soluble probes that do not interact with intestinal nutrient transporters, L-arabinose (molecular weight M_r_ = 150.1 Da) and lactulose (M_r_ = 342.3) [25]. As a comparator, we also measured absorption of 3-O-methyl-D-glucose (M_r_ = 194.2), which is a nonmetabolized analogue of D-glucose that is passively absorbed through the paracellular space but also actively transported across the enterocyte membrane by the Na^+^-glucose cotransporter SGLT1. Using these probes we tested three specific predictions: (1) absorption of L-arabinose would exceed that of lactulose, because the paracellualr pathway discriminates according to molecule size; (2) absorption of these two paracellular probe molecules would be higher in the avian than rodent species; and (3) absorption of the D-glucose analogue would be complete in all the animals because mammals and birds possess intestinal SGLT1 [29].

## Materials and Methods

### Ethics statement

The animal care and experimental procedures were approved by the Committee of Animal Experimentation of the University of Haifa (Permit number 096/08) and the Animal Care and Use Committee at the University of Wisconsin College of Agricultural and Life Sciences (Protocol A01355-0-06-08).

### Animals and their maintenance

Animals were trapped at various locations in Israel under permit from the Israel Nature and National Parks Authority: Common/Egyptian spiny mouse, *Acomys cahirinus* (m_b_ = 57.9±2.1 g, *n* = 13); Golden spiny mouse, *Acomys russatus* (m_b_ = 58.0±2.5 g, *n* = 6); Tristram's grackle, *Onychognathus tristramii* (m_b_ = 111.3±2.4 g, *n* = 9); Yellow-vented bulbul, *Pycnonotus xanthopygos* (m_b_ = 35.8±3.9 g, *n* = 7). Rodents were housed in pairs in plastic cages (21×31×13 cm) under relatively constant environmental conditions (25±2°C, relative humidity of 35±3%), a lighting schedule of 12∶12 light∶dark, and had access to *ad libitum* rodent chow (Koffolk serial no. 19510) and fresh carrots as a source of free water. Birds were housed in a large open outdoor cage and maintained *at libitum* water, fruits (apples, grapes, melons), vegetables (tomatoes, cucumbers), dog chow (Bonzo serial no. 651410), and chopped boiled eggs. For the experiments the birds were transferred to individual cages (40 cm×40 cm×60 cm) in a room under the same environmental conditions as described for rodents. All animals were acclimated to the laboratory for at least a week before they were used in experiments.

### Test compounds

Carbohydrates were purchased from Sigma Chemicals (St. Louis, MO, USA): L-arabinose (M_r_ = 150.1), D-glucose (M_r_ = 180.2), 3-O-methyl-D-glucose (3OMD-glucose; M_r_ = 194.2), lactulose (M_r_ = 342.3). Radiolabeled chemicals were purchased from American Radiolabeled Chemicals Inc. (ARC, St. Louis, MO, USA, through Ornat Biochemicals & Laboratory Equip. Ltd, P.O.B 2071 Rehovot 76120, Israel): Arabinose, L-[1-14C], 50 µCi , 0.1 mCi/ml, Lactulose, [D-galactose 6-3H], 250 µCi , 1 mCi/ml, Methyl-D-glucose, 3-0-[methyl-3H], 1 mCi , 1 mCi/ml, all 9∶1 in ethanol∶water.

### Fractional absorption of probes measured *in vivo*

As a measure of passive, paracellular absorption, we used standard methods from pharmacokinetics to measure the whole-organism fractional absorption of water-soluble compounds. As described in more detail, below, metabolically inert probe molecules were injected and also administered orally with a gavage needle to intact animals in separate experiments, and blood and/or urine samples were serially collected and analyzed for the probe molecules by liquid scintillation. Fractional absorption (*f*) was calculated as [post oral administration AUC]/[ post injection AUC] where AUC = dose-corrected area under the curve of plasma or urine probe amount vs. time. This simple pharmacokinetic method does not require assumptions about pool sizes (e.g., 1 or 2 pools) or kinetics (e.g., 1^st^ order) [30]. In mammals, the probes can be recovered in urine, and estimates of oral absorption take account of possible differential recovery by incorporating data on probes when injected. As shown in Results, recoveries of carbohydrate probes were uniformly high, which is in agreement with measures by others in rats and humans [31]. In birds, plasma is sampled rather than urine, because urine is mixed in the avian cloaca with undigested residue from the intestine. Fractional absorption measured in laboratory rats by serially sampling blood did not differ significantly from that measured by urine recovery for L-rhamnose, another metabolically inert carbohydrate probe [26], but this comparison has not been performed for the probe molecules we used. In Discussion we elaborate on comparisons between these two procedures and reasons to choose them.

Food was withheld during the animals' 12-hour normal inactive period just preceding the measurement period during their normal activity period. Also, the rodents were transferred to metabolic cages with wire bottoms and a tray beneath from which to collect clean urine samples. At the beginning of a trial, animals were orally dosed at 1.0% body mass with an isosmotic solution containing L-[^14^C]arabinose (∼0.02 µCi/g animal) and either [^3^H]3OMD-glucose (∼0.1 µCi/g animal) or [^3^H]lactulose (∼0.5 µCi/g animal). Oral dose solutions also contained NaCl (∼90 mM, ∼180 mOsm) and D-glucose (∼90 mM, ∼90 mOsm). NaCl was included in the solution to balance osmolality. Inclusion of Na^+^ also provides an essential ion for Na^+^-coupled D-glucose absorption, although it is not strictly necessary in this kind of whole-animal study because animals would still absorb nearly all glucose even if the diet is low in Na^+^ because additional Na^+^ is secreted into the intestinal lumen together with bicarbonate and diffuses from blood [32]. In a separate experimental trial, each animal was injected (0.6% body mass) with isosmotic NaCl solution containing L-[^14^C]arabinose (∼0.02 µCi/g animal) and either [^3^H]3OMD-glucose (∼0.1 µCi/g animal) or [^3^H]lactulose (∼0.1 µCi/g animal). The injection site was the pectoralis muscle in birds and the peritoneal cavity in rodents. Syringes were weighed before and after dosing animals to determine actual dose administered.

After administration of radiolabeled probes, birds and rodents were returned to their cages, where birds had *ad libitum* access to fruit and the rodents had *ad libitum* access to sucrose solution (10% w/w). The purpose was to provide rodents water and some calories and also make them urinate more [33]. Serial blood samples (∼25 µL) were drawn from the brachial vein of birds as described previously [34] at t = 0 (background), 10, 20, 30, 45, 60, 80 or 90, 120, 150 min post-injection or post-oral administration. Plasma was separated using a standard hematocrit centrifuge, placed in scintillation vials and weighed. Cages of rodents were checked for urine collection beginning 30 min after probe administration and every 30-min thereafter until 6 hours, and then at 2-h intervals thereafter. Urine samples were placed in scintillation vials and weighed. Scintillation cocktail (Quicksafe A, Zinsser, UK) was added and samples were counted for disintegrations per minute (dpm) by liquid scintillation (Packard Tri-Carb 1600 TR scintillation counter, Packard INC., Meriden CT, USA), with corrections for quench and appearance of ^14^C counts in the ^3^H channel.

### Analysis of data on urinary excretion by rodents

For each compound, the amount (dpm) in total urine volume at each sample time *t* was normalized to the respective dose and multiplied by 100. These values for % of dose, and also cumulative % of dose were plotted as a function of *t*. The cumulative % recovery (*CPR*) post-injection was compared with 100% (i.e., total recovery) using the 95% confidence interval. Recoveries post-injection were high but not always complete (i.e., <100%) (see Results). Consequently, the fractional absorption for orally administered probes was calculated as
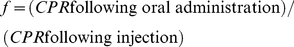
(1)


### Analysis of data on plasma of birds

For each compound, the concentration (dpm) in each plasma sample at time *t* was normalized to the weight of each sample (*C_t_*, dpm mg^−1^ plasma) and to the animal's respective dose and then plotted against sampling time since the compound was administered either orally or by injection. The integration of the area under this curve (*AUC_t_*) represents the amount of compound that has been absorbed from time 0 up to time *t*, whereas *AUC_total_* denotes the total amount of compound absorbed from 0 up to infinity time (∞). Following typical procedures in pharmacokinetics [35], the area from *t* = 0 to *t* = *x* min (when the final blood sample was taken) was calculated using the trapezoidal rule. The area from *t* = *x* min to *t* = ∞ was calculated as *AUC* ^x→∞^ = *C_t_* (at *t = x*)*/k*, where *k* is a rate constant which was typically determined for each bird based on slope of the terminal portions of its curves post injection and post absorption. The total *AUC* ^0→∞^ was obtained by summing the two areas. Fractional absorption (*f*), or bioavailability, for each compound was estimated based on the ratio between the area under the plasma concentration versus time curve for oral administration experiments (*AUC_oral_*, in units of dmp·min·mg plasma^−1^) and injection experiments (*AUC_inj_*) normalized to respective dosage given to the animal:
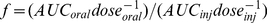
(2)Both methods (eq.s 1 and 2) of calculating *f* are favored because they make no major assumptions about compartments or kinetics. Fractional absorption estimates how much of the ingested probe was absorbed into the animal's system. The calculations of *f* and their statistical comparison (below) were performed based on data for individuals, although data shown in figures are mean values corrected for differences in dose between individuals, plotted against approximate sampling time bins.

### Statistical analysis

Numerical data are presented as means ± SEM (*n* = number of animals). Although data shown in the figures are mean values, statistical analyses were performed based on data for individuals. Fractional absorption (*f*) and other proportional data were arcsine square root transformed before statistical analysis [36]. Results were analyzed by ANOVA, repeated measures ANOVA, ANCOVA, and Student's t-test. Nonlinear curve fitting (Gauss-Newton algorithm in SYSTAT) was used to fit kinetic data, and kinetic model fits were compared according to [37]. The following one- and two-compartment models were compared when analyzing the curves of *C_t_* vs. sampling time post-injection for the birds:
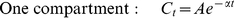
(3)

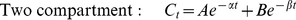
(4)where values of *A* and *B* are the inverse of apparent pool sizes, and *α* and *β* are the respective elimination rate constants from the pools. Statistical significance was accepted at *α*<0.05. One-tailed tests were used for *a priori* predictions.

## Results

### Recovery of injected probes in rodents

**Figure 1:**
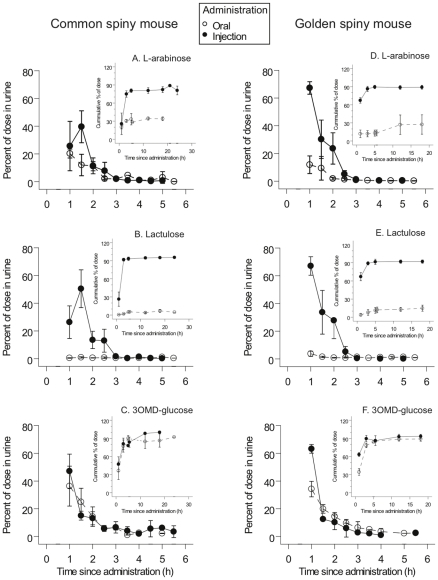
Urinary elimination of probe molecules as a function of times since administered to rodents. Empty circles denote probe in urine following oral administration; filled circles represent probe in urine after administration by injection. The left column (panels A, B, and C) shows plots for common spiny mice for, respectively, L-arabinose, lactulose, and 3OMD-glucose; the right column (panels D, E, and F) show the respective plots for golden spiny mice. Each pair of plots shows the percent of the dose eliminated as a function of time through the first six hours and the cumulative % of dose eliminated over the full 18-hour trial (the inset, with a smaller number of data points). Error bars are SEM, and sample sizes were 4–6 mice in each case.

The vast majority of probes that were injected were recovered in urine within three hours post-injection (Fig. 1). Recoveries of probes injected into *A. cahirinus* were: L-arabinose, 84±5% (*n* = 4; Fig. 1A); L-lactulose, 96±2% (*n* = 4; 1B); 3-*O*-methyl-D-glucose, 100±1% (*n* = 4; 1C). Recoveries for *A. russatus* were, respectively: 89±3% (*n* = 6; Fig. 1D); 92±2% (*n* = 6; 1E); 93±3% (*n* = 4; 1F). Post-injection recoveries did not differ significantly between species (*F*_1,22_ = 0.34, *P*>0.5) but did differ significantly among the probes (*F*_2,22_ = 6.11, *P* = 0.008), with no significant species X probe interaction *F*_2,22_ = 2.27, *P*>0.1). When the data for species were pooled for each probe, recoveries were significantly less than 100% for both arabinose (95% C.I. 81–93%, *n* = 10) and lactulose (95% C.I. 90–97%, *n* = 10) but not for 3-*O*-methyl-D-glucose (96–101%, *n* = 8). Consequently, fractional absorptions by individuals of orally administered probes were corrected for incomplete recovery by dividing them by the respective species mean recovery post-injection using eq. 2 in Methods.

### Absorption of orally administered probes by rodents

The time course for recovery of orally administered probes was very similar to that for injected probes (Fig. 1). Fractional absorptions differed significantly among the probes (*F*_2,21_ = 98.5, *P*<0.001) but did not differ significantly among the rodent species (*F*_1,22_ = 0.003, *P*>0.9), with no significant probe X species interaction (*F*_2,21_ = 1.62, *P*>0.2; Fig. 1). In both rodent species 3-*O*-methyl-D-glucose was almost entirely absorbed (0.95±0.01, *n* = 7; Fig. 1C,F), but fractional absorptions were considerably lower for both arabinose (0.37±0.06, *n* = 10; Fig. 1A,D) and for lactulose (0.085±0.021, *n* = 10; Fig. 1B,E). Each mean value for a probe differed significantly from that of the other probes.

### Absorption of orally administered arabinose by birds

**Figure 2:**
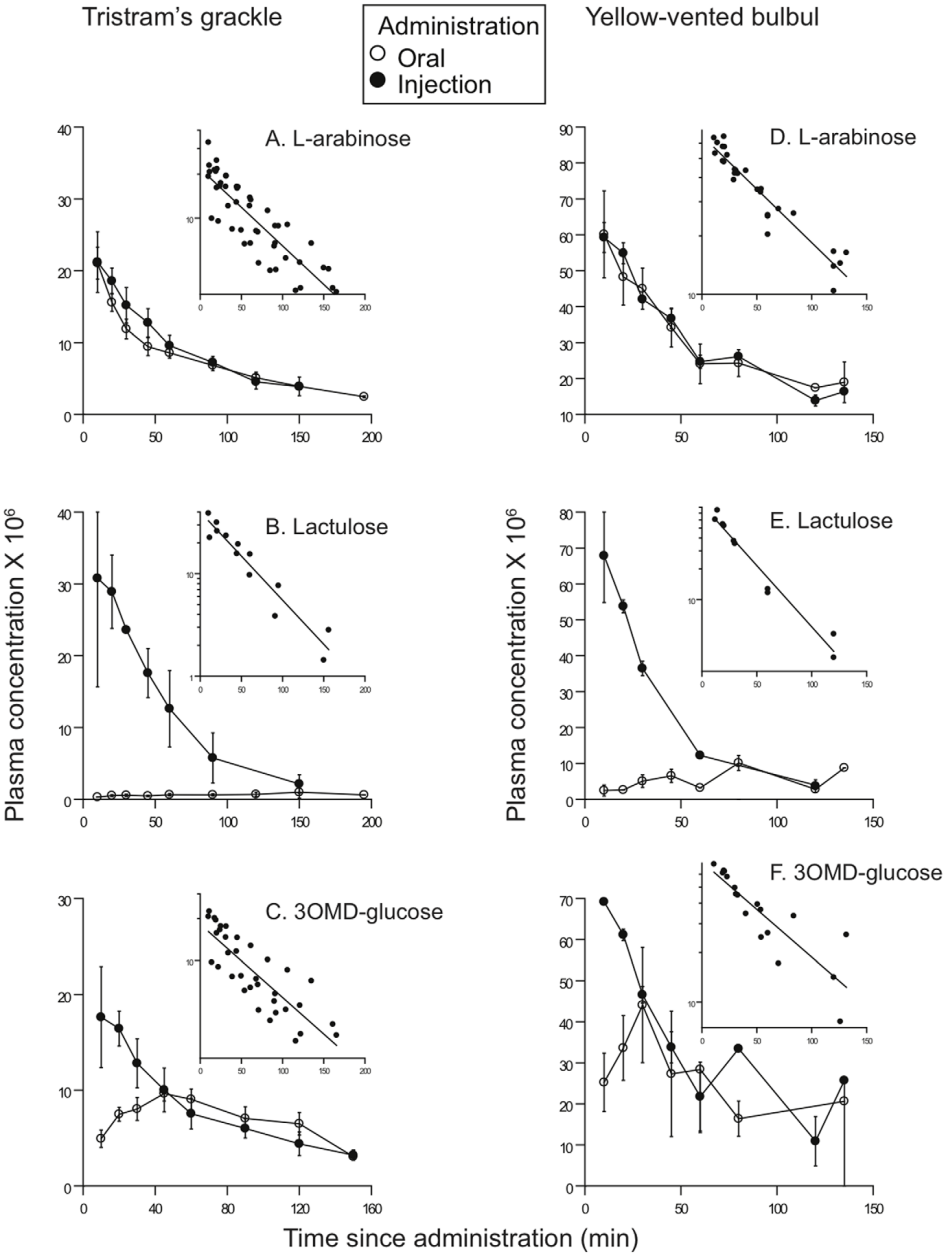
Plasma levels of probe molecules as a function of time since administered to birds. Empty circles denote probe in plasma following oral administration; filled circles represent probe in plasma after we administered by injection. The left column (panels A, B, and C) shows plots for Tristram's grackle for, respectively, L-arabinose, lactulose, and 3OMD-glucose; the right column (panels D, E, and F) are the respective plots for yellow-vented bulbuls. Each pair of plots shows the plasma concentration normalized to dose (multiplied by 10^6^ for ease of display) as a function of time and the semi-log plot of plasma values post-injection, fit to a line by linear regression (the inset). Bars are SEM, and sample sizes were 4–7.

Absorption was apparently rapid because plasma samples taken at 10 min post oral in both species were already maximal (no initial rise), as was also the case in injection experiments (Figure 2A,C). Terminal slopes in oral trials, based on the last 4 points in grackles and the last 3 points in bulbuls, did not differ significantly from those in injection trials in either grackle (*n* = 5, paired test *t* = 1.09, *P*>0.3) or bulbul (*n* = 6, paired test *t* = 1.86, *P*>0.15). More blood samples were taken from the larger grackle, which explains why more time points were used to calculate terminal slopes in that species. AUC_oral_ was measured in 8 grackles using the terminal slope from the oral trial itself (6 cases), the terminal slope from the injection trial on that same individual (1 case), or the mean terminal slope for all oral trials (1 case). For two grackles, a single point at 50-min was estimated based on interpolation of the other points for those individuals and the general pattern among all birds. AUC_oral_ was measured in 7 bulbuls using the terminal slope from the oral trial itself (6 cases) or the mean terminal slope for all oral trials (1 case). Arabinose fractional absorption (*f*) was complete in both species, averaging 0.98±0.07 (s.e., *n* = 8) in grackle and 1.10±0.15 (s.e., *n* = 7) in bulbul (*t*_13_ = 0.8, *P*>0.4).

### Absorption of orally administered lactulose by birds

In both species, lactulose absorption was slow based on very low counts in plasma post oral administration, compared with plasma counts post injection, and the absence of terminal slopes in the oral trials (Figures 2B,E). Consequently, AUC_oral_ was measured using the terminal slope from the injection trials, which could result in a small underestimate of *f*. But, *f* was clearly very low, averaging 0.069±0.011 (*n* = 4) in grackle and 0.30±0.10 (*n* = 3) in bulbul (*t*_5_ = 3.45, *P* = 0.018).

### Absorption of orally administered 3-O-methyl D-glucose (3OMDG) by birds

Absorption was apparently not as rapid as for arabinose, because plasma 3OMDG did not peak until 30 min post oral administration for bulbuls and 40–50 min post oral for grackles (Figure 2C,F). Terminal slopes in oral trials did not differ significantly from those in injection trials for either grackle (*n* = 4, paired test *t* = 0.42, *P*>0.7) or bulbul trials (*n* = 4, paired test *t* = 0.16, *P*>0.8). AUC_oral_ was measured in 5 grackles using the terminal slope from the oral trial itself (all cases), although for one grackle a single point at 50-min was estimated based on interpolation of the other points for that individual and the general pattern among all grackle. AUC_oral_ was measured in 4 bulbuls using the terminal slope from the oral trial itself (all cases). 3OMD-glucose fractional absorption (*f*) was complete in both species, averaging 0.95±0.08 (*n* = 5) in grackle and 0.80±0.19 (*n* = 4) in bulbul (*t*_7_>0.8, *P*>0.4).

### In summary for birds

Absorption of both arabinose and 3OMDG by birds was complete (not significantly different from 1.0). As expected, lactulose absorption was lower. In a two-way ANOVA (factors = probes and species), there was a significant difference among the probes in *f*, but no significant difference among the species (no significant interaction either between probe and species). Hence, we pooled the data by species, and performed a one-way ANOVA for differences among probes. There was no significant difference in *f* between arabinose (1.03±0.07, *n* = 15)) and 3OMG (0.88±0.08, *n* = 9), but *f* for lactulose (0.17±0.06, *n* = 7) was significantly lower than for both other probes (P<0.001 in both cases).

### Comparison of fractional absorptions among birds and rodents

**Figure 3:**
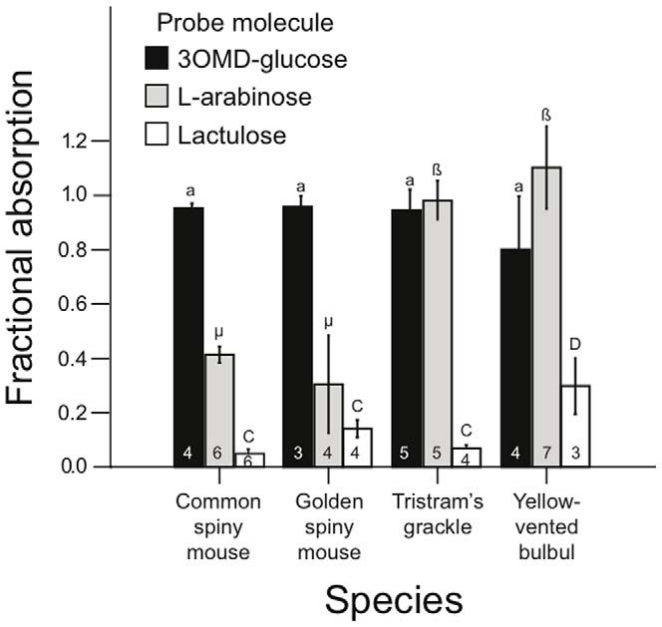
Comparison of fractional absorption of three probe molecules among four species. Means and SEM are shown, and sample sizes are the numbers within each of the bars. For each probe type, where bars are accompanied by common letters, the means did not differ significantly based on statistical analyses described in Results. Thus, all species absorbed 3OMD-glucose to the same high extent, arabinose absorption was significantly higher in the bird species than in the rodent species, and lactulose absorption was low in all the species but, among them, slightly higher in yellow-vented bulbuls.

The four species did not differ significantly in *f* of 3-O-methyl-D-glucose (*F*_3,16_ = 0.65, *P*>0.5), all absorbing nearly 100% (Fig. 3) Both avian species, however, had significantly higher *f*s of L-arabinose than did the two rodent species (*F*_3,21_ = 13.9, *P*<0.001). Fractional absorption of lactulose was significantly higher in the bulbul than in the other three species (*F*_3_,_13_ = 11.1, *P*<0.001), which did not differ significantly from each other.

### Comparison of rates of absorption by birds

**Figure 4:**
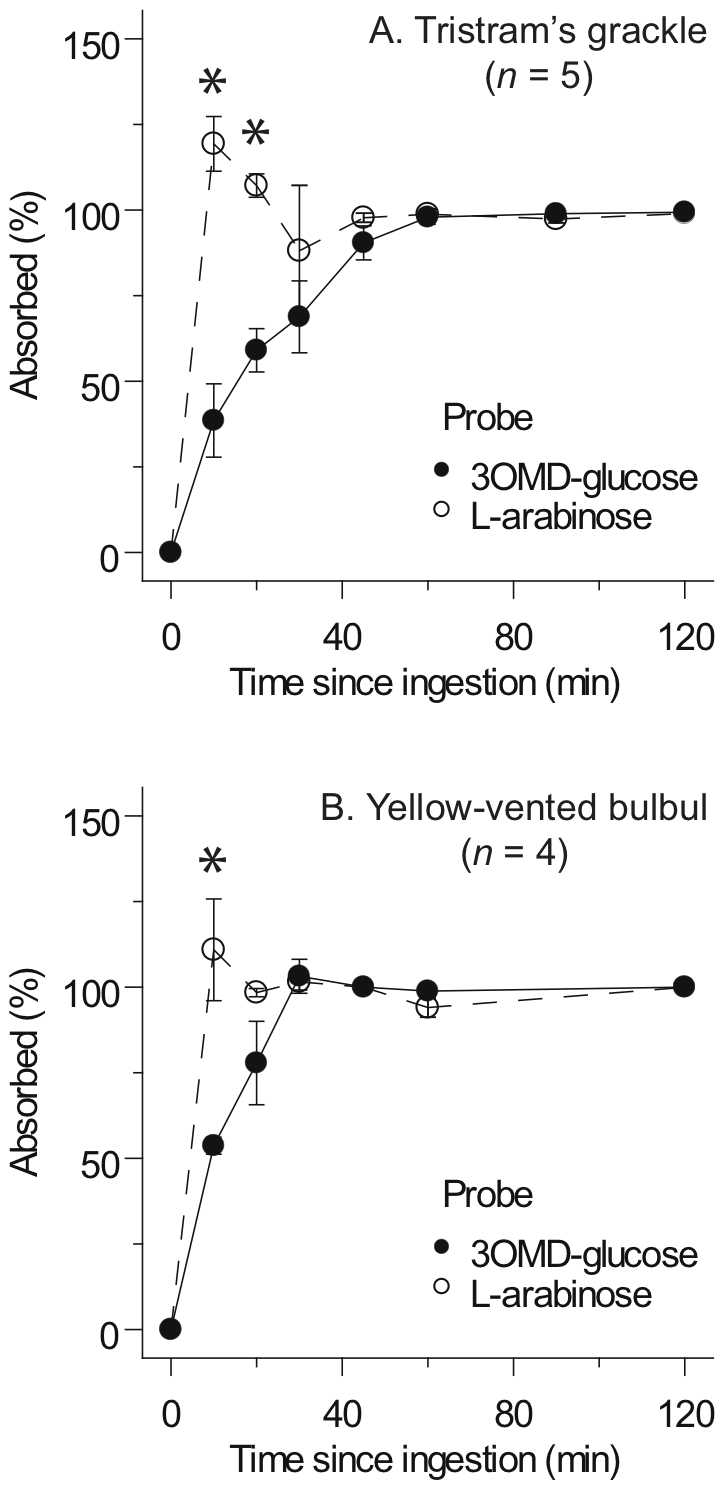
Cumulative absorption versus time since probe ingestion. Data for 3-O-methyl-D-glucose (3OMD-glucose) are denoted by filled circles and solid lines; L-arabinose data are represented by empty circles and broken lines. Cumulative absorption data for Tristram's grackles and yellow-vented bulbuls are shown in panels A and B, respectively. By an hour or less post-ingestion, the absorption of both compounds was essentially complete, but at early time points the absorption of L-arabinose significantly exceeded that of 3OMD-glucose, as indicated by an asterisk (P<0.05; paired t-test). Values are means (± SEM) calculated from measurements on individual animals.

The time course over which absorption of the carbohydrates occurred was derived by the method of Loo and Riegelman [38], which requires information on kinetic constants derived from the injection plots (Table 1) and the mean plasma concentrations following oral administration of each compound (Figs. 2 and 3). Semi-log plots of injection data (insets, Figs. 2 and 3) were not significantly better fit by a model of bi-exponential than mono-exponential decline for either L-arabinose or 3OMD-glucose, and r^2^ values for the fits of the two carbohydrates to the mono-exponential model in both species were all >0.98; Table 1). Using these values, the time course (i.e., rate) of absorption for L-arabinose and 3OMD-glucose were compared for individuals in which absorption of both were measured simultaneously (Figure 4). Significantly more L-arabinose was absorbed, compared with 3OMD-glucose, at 10-min post-ingestion in both species (paired test *t*>4.4; *P*<0.05), and still at 20-min post-ingestion in the grackle (*t*_2_ = 7.2, P = 0.02).

**Table 1:** Parameters for mono-exponential fit of dose-corrected plasma probe concentrations from injection experiments.

	Tristram's grackle		Yellow-vented bulbul	
Probe parameter	L-arabinose	3OMD-glucose	L-arabinose	3OMD-glucose
	(*n* = 7)	(*n* = 5)	(*n* = 6)	(*n* = 4)
A (mg^−1^ plasma)	25.1±3.1	22.6±3.0	73.1±4.2	81.1±10.4
α (min^−1^)	−0.014±0.002	−0.016±0.002	−0.012±0.001	−0.016±0.005
r^2^ values	≥0.994	≥0.983	≥0.989	≥0.988

Data here are a summary of that shown in Fig. 2, and are mean ± SEM.

The model fit for each bird was *C_t_* = *A e^−αt^* (see Eq. 3, Methods).

3OMD-glucose = 3-O-methyl-D-glucose.

## Discussion

To measure fractional absorption, we used a simple pharmacokinetic method (eq. 1 or eq. 2) that does not require assumptions about pool sizes (e.g., 1 or 2 pools) or kinetics (e.g., 1^st^ order) [30]. Probes molecules that are absorbed are ultimately filtered by the kidney and eliminated in urine if they are metabolically inert, as illustrated by the near-quantitative recovery in the rodents' urine of all three probes. Our methods (eq. 1 and 2) also took account of possible differential recovery by incorporating data on probes when injected. In birds, plasma is sampled rather than urine, because urine is mixed in the avian cloaca with undigested residue from the intestine, but in principle the methods reflected in equations 1 and 2 should yield similar estimates of fractional absorption [39], [40]. For the rodents we collected urine rather than blood out of concern for their welfare, because in such small rodents urine collection seems less traumatic than repeated blood sampling via puncture of the retro-orbital region or of the saphenous vein [41] or tail vein. In larger rats we have compared the two methods in measuring fractional absorption of L-rhamnose and, as expected from theory, the estimate from serially sampling blood (0.24±0.03) did not differ significantly from that measured by urine recovery (0.22±0.03, n = 4, by repeated measures F_1,3_ = 1.14; p>0.3) [26]. However, the measurement by urine recovery, unlike blood sampling, does not lend itself to calculations of rate of absorption, and so we could analyze and discuss rates only in the birds.

### Prediction 1: absorption of L-arabinose will exceed that of lactulose

Because the paracellular pathway discriminates according to molecule size, due to sieving at the tight junction [19], [42], we predicted that absorption of L-arabinose would exceed that of lactulose, which is what we found in each of the four species. Like other mammals and birds, absorption of water-soluble neutral probe molecules that are not actively transported diminishes considerably as molecule M_r_ approaches 400 Da [43], which corresponds to a molecular radius of about 5 angstroms [19]. We studied absorption of neutral probes, but the tight junction is cation selective for molecules in the size range we measured [20], [21] and so rates of absorption could be higher for positively charged molecules. Rates of absorption are also typically higher when measured in the presence (*vs.* absence) of luminal nutrients [13], [19], [44], [45], which is why we included 90 mM D-glucose in the solutions we administered orally and why we provided access to food or glucose solution during trials.

These factors of molecule size, charge, and associated nutrients help define the conditions under which water-soluble SMs may be best absorbed. A number of studies, mainly using intestinal tissue in cell culture, have demonstrated paracellular absorption of alkaloids [23], [46] and phenolics [47], [48], [49], [50], [51] in the M_r_ range 162–460 Da, but there are few measurements in intact animals. Although cell culture studies are useful for demonstrating the potential for absorption, whole animal studies complement them by establishing an absolute capacity that can be interpreted in terms of likely nutritional significance. The whole-animal studies of paracellular absorption support the notion that smaller water soluble SMs that have been shown to be absorbed by the paracellular path in cell culture, such as nicotine (M_r_ 162.2; [23]), gallic acid (M_r_ 170.1; [52]), caffeine (M_r_ 194.2; [46]) and catechin (M_r_ 290.3; [47]), might be absorbed in substantial amounts in some animals. For example, based on knowledge of the MW size-dependence of paracellular absorption in rock doves [25], we (ECVand WHK) predicted that they would absorb around 40% of a dose of nicotine in its cationic, water soluble, form. Using our typical pharmacokinetic approach and “area under the curve” calculations, we found that rock doves absorbed 44±3% (*n* = 6; F. D. Cid, E. Caviedes-Vidal and W.H. Karasov, unpubl. observations). However, the larger size of esterified phenolics such as chlorogenic and rosmarinic acids (M_r_ 354–364; [49], [51], [53]) and the glycoside myricitrin (M_r_ 464 Da; [47], [50]) might preclude much paracellular absorption of those compounds.

### Prediction 2: Absorption of paracellular probe molecules will be higher in the avian than rodent species

Quantitatively, paracellular absorption is at least twice greater in small birds (<400 g) than in non-flying mammals [26]. Fractional absorption (*f*) of L-arabinose in our birds was essentially complete, compared with values of 0.61 in both house sparrows [54] and rock doves [25], but values are lower in all non-flying mammals studied, including our rodents (*f* = 0.37), laboratory rats (*f* = 0.34; [25]) and humans (*f* = 0.26 when corrected for incomplete urine recovery [55]). Notably, the differences in paracellular absorption between our avian and rodent test species did not extend to absorption generally, because whereas the rodents had relatively low fractional absorption of L-arabinose, their absorption of 3-O-methyl-D-glucose, was high and not significantly different from that of birds. The difference in paracellular absorption between the birds and the rodents is not explained by longer retention of digesta in the gut of the former relative to the latter, because avian species typically have shorter mean retention time of digesta than do similar sized mammalian species [56]. In our case also, retention times of the birds (grackles, 135 min; bulbuls, 35 min; [28]) were much shorter than those of the rodents (golden spiny mouse, 9 h; common spiny mouse, 6 h; M.S-B, unpublished data). Because birds typically achieve higher paracellular absorption with less intestinal length and surface area than do similar sized non-flying mammals [26], there apparently are differences in intestinal permeability per unit intestinal tissue. Indeed, under similar recirculating duodenal perfusion conditions, anesthetized rats and pigeons absorbed D-glucose at a comparable rate but the pigeons had significantly greater (>2× higher) absorption of inert carbohydrate probes per unit intestine length or area [25].

The ecological significance of the difference between small birds and mammals in paracellular absorption will likely depend also on other features of their physiology. Concentrations of SMs at tissues, or target site(s) if the SMs are toxic, may depend on permeability of other barriers and also on relative rates of elimination, which are determined by rates of biotransformation and kidney glomerular filtration rates and/or biliary excretion of the parent compound and its metabolite(s). We do not know of a direct comparison between mammals and birds in overall biotransformation rates, but the increased reliance on the paracellular pathway in birds hints at a tradeoff: the free energy birds gain by absorbing nutrients passively may be offset by the metabolic demands placed on them to biotransform and eliminate concomitantly absorbed SMs. As for glomerular filtration, we also do not know of a direct comparison between these two groups of animals, although allometric summaries are available for both birds [57] and mammals [58].

### Prediction 3: Absorption of the D-glucose analogue would be complete in all the animals

Active transport of D-glucose, which occurs via SGLT1 in both mammalian and avian intestine [29], provides an excellent mechanism to absorb remaining glucose in the gut even at very low concentrations. Hence, there is the expectation that most glucose will be absorbed, which is what we observed. But, the extent to which glucose is absorbed actively via intestinal SGLT1 *vs*. passively via paracelluar absorption differs between mammals and birds, and this has implications for interactions with SMs also, as we discuss below. The relative extent of passive glucose absorption has been effectively studied in avian and mammalian species by measuring simultaneously the absorption of D-glucose (or its nonmetabolizable analogue 3OMD-glucose) and the absorption of a water-soluble probe whose absorption is not mediated by any membrane transporter. For example, based on simultaneous measures with D-glucose and L-glucose (the stereoisomer not actively transported), L-glucose can account for the majority (range 50 to >90%) of glucose absorption in two avian species [59], [60]. But, in analogous studies in rats [61], dogs [62], and humans [63], L-glucose, and hence passive absorption, is quantitatively much less important.

To estimate how much absorption of 3OMD-glucose was passive in our test species, we assumed that absorption of L-arabinose is a proxy for passive absorption of 3OMD-glucose, once adjusted for the small difference in MW. Because diffusion in water declines with MW^1/2^ [64], each value of L-arabinose absorption was decreased by 12% ( = 100*[194^1/2^–150^1/2^]/194^1/2^). Assuming that the absorption of 3OMD-glucose represents the sum of passive+mediated absorption, the ratio of the amounts absorbed (L-arabinose/3OMD-glucose) indicates the proportion of 3OMD-glucose absorption that occurs via the passive pathway. The ratios of *f*_L-arabinose_/*f*_3OMD-glucose_ (corrected for difference in MW), was 1 in the birds and 0.34 in the rodents. Clearly, the vast majority of 3OMD-glucose absorption was apparently passive in the birds but not in the rodents. However, a ratio of 1 seems implausible if 3OMD-glucose is absorbed in the birds simultaneously by the active and passive processes. Likewise, the apparently more rapid absorption of arabinose compared with 3OMDG, indicated by the lack *vs.* presence of an absorption phase in the plasma concentration curves of L-arabinose *vs*. 3OMD-glucose, respectively (Fig. 2), and as depicted also in Figure 4, seemingly cannot be explained based on differences in diffusion due only to MW differences. An explanation may be that paracellular absorption is very high in the birds and that there is a large effect of sieving at the paracellular junction, which slows down passive absorption of the larger 3OMD-glucose molecule relative to the smaller L-arabinose, so that absorption of the latter is completed sooner. Essentially, the fast paracellular absorption of arabinose, due to its smaller size compared with 3OMD-glucose, balances the positive effect of active transport for 3OMD-glucose. It is clear from these findings that passive paracellular absorption is very rapid in the birds, notably so for smaller water-soluble compounds, and that this pathway also would provide a rapid and energetically cheap route for absorption of amino acids and sugars that would complement their other transporter-mediated and/or active routes of absorption.

Some plant SMs have the potential to reduce mediated glucose transport, and the difference between small birds and nonflying mammals in reliance on paracellular absorption might lead to different sensitivities to their effects. Flavonoids are a class of polyphenolic compounds ubiquitous in higher land plants that have numerous biological effects including, in some cases, inhibition of glucose absorption by glucose transporters [65]. The most famous examples are phloretin and phloridzin, which are used by membrane physiologists as inhibitors of GLUT-2 and SGLT-1 respectively, in glucose absorption studies with isolated tissue, cell culture, and membrane vesicles. Skopec et al. (2010) predicted that phloridzin would inhibit glucose absorption at the whole animal level when administered at ecological concentrations (they used 10 mM), and that the effects would be more pronounced in nonflying mammals that rely on mediated pathway(s) for glucose absorption than in birds that rely more on a nonmediated, paracellular pathway. They found that phloridzin inhibited whole-animal glucose absorption efficiency by >36% in laboratory rats, whereas it did not significantly decrease glucose absorption in American robins [65]. Another flavonoid, isoquercetrin, also significantly decreased glucose absorption in rats but not in robins [65]. They did not ascribe the difference to any major difference between rat and robin in the types of intestinal glucose transporters, because birds and mammals appear to share the similar suite of intestinal sugar transporters [66], [67]. Instead, they ascribed the difference in the inhibition by these plant secondary metabolites of glucose absorption to the rats' much greater reliance on glucose transporters for intestinal glucose absorption than is the case for robins.

### Ecological implications of differences in physiological processing of SMs

Because plant SMs mediate so many interactions between mammals and birds and their plant resources (e.g., leaf, fruit and seed diet selection, seed and pollen dispersal), physiological differences between mammals and birds in their responses to SMs should have many ecological ramifications [68]. There are other examples of physiological differences between mammals and birds that may have ecological significance [69]. Whereas some mammals may be more likely to chew and digest seeds within fruits, frugivorous birds such as the grackles and bulbuls may be more likely to egest the seeds and be seed dispersers. The Directed Toxicity Hypothesis [68], [70] states that SMs in ripe fruit are toxic (or deterrents) for vertebrate fruit consumers that do not disperse viable seeds, but have no or little toxic effect on seed-dispersing frugivores [71], [72], [73]. The sensation(s) induced by irritants such as capsaicin deters mammals but not birds [73]. There is some evidence that birds and mammals have molecular differences in the vanilloid receptor, and thus different sensations mediated by vanillan receptor [74]. Struempf et al. [75] found that the cyanogenic glycoside amigdalin, a compound toxic to some mammals, does not deter fruit consumption by frugivorous cedar waxwings, probably because they lack the capacity to hydrolyze it. As is the case for most of these examples, the full ecological implications of the differences between mammals and birds in paracellular absorption remains to be explored.
